# The development of contour processing: evidence from physiology and psychophysics

**DOI:** 10.3389/fpsyg.2014.00719

**Published:** 2014-07-08

**Authors:** Gemma Taylor, Daniel Hipp, Alecia Moser, Kelly Dickerson, Peter Gerhardstein

**Affiliations:** ^1^Department of Psychology, Binghamton University, State University of New YorkBinghamton, NY, USA; ^2^US Army Research Laboratory, Department of the Army, RDRL-HRS-D, Aberdeen Proving GroundsMD, USA

**Keywords:** contour detection, closure, horizontal connections, development, visual development

## Abstract

Object perception and pattern vision depend fundamentally upon the extraction of contours from the visual environment. In adulthood, contour or edge-level processing is supported by the Gestalt heuristics of proximity, collinearity, and closure. Less is known, however, about the developmental trajectory of contour detection and contour integration. Within the physiology of the visual system, long-range horizontal connections in V1 and V2 are the likely candidates for implementing these heuristics. While post-mortem anatomical studies of human infants suggest that horizontal interconnections reach maturity by the second year of life, psychophysical research with infants and children suggests a considerably more protracted development. In the present review, data from infancy to adulthood will be discussed in order to track the development of contour detection and integration. The goal of this review is thus to integrate the development of contour detection and integration with research regarding the development of underlying neural circuitry. We conclude that the ontogeny of this system is best characterized as a developmentally extended period of associative acquisition whereby horizontal connectivity becomes functional over longer and longer distances, thus becoming able to effectively integrate over greater spans of visual space.

## INTRODUCTION

The early visual system is one of the first avenues by which infants begin to learn about the world around them (Piper and Darrah, 1994). Visual capabilities begin developing before birth (Alberts, 1984), undergo considerable maturation in the first few months after birth (Johnson, 2001; Lewis and Maurer, 2005; Atkinson and Braddick, 2007), and continue into adolescence (see Slater and Johnson, 1998; Pennefather et al., 1999; Hadad et al., 2010b). Visual development has been characterized with varying degrees of specificity across several domains, including: sensitivity to spatial frequency (Patel et al., 2010), orientation (Braddick et al., 1986; Morrone and Burr, 1986; Candy et al., 2001), motion (Johnson, 2001; Wattam-Bell et al., 2010), color perception (Bornstein et al., 1976; Gerhardstein et al., 1998), and facial recognition (Bushnell et al., 1989; Johnson et al., 1992; for a recent review, see Braddick and Atkinson, 2011). However, many descriptions of the mechanisms through which infants begin to make sense of their visual world and how these mechanisms might change across ontogeny are somewhat sparse.

The goal of the present paper is to review, discuss, and integrate findings from across infancy and childhood in order to shed light on the development of contour detection and integration from first emergence to adult-level function. Throughout this review, psychophysical data will be augmented by data from physiological and theoretical studies, and adult data will be used to inform the examination of the developmental path where possible. We will focus on how the visual pathway implements initial contour processing across development. Therefore, we will not discuss the role of top-down processing in modulating object perception in depth, as that topic is beyond the scope of this review. We conclude with a discussion of how to interpret what appears to be quite protracted unfolding of this system, and with a call to action for further research in areas where data is lacking.

## PATH TO OBJECT PERCEPTION

Construction of a clear and meaningful percept of a visual scene is a demanding computational problem. Developing basic acuity in infancy and orientation sensitivity (Banks and Salapatek, 1981; Morrone and Burr, 1986; Sireteanu et al., 1994; Candy et al., 2001) is an important first step toward the development of pattern and object perception in the visual world (see also Wattam-Bell et al., 2010). Detecting regions within the visual field that contain points of locally high contrast and then integrating these early representations into a contour-level description of the scene (e.g., Marr, 1982) can then be used to infer object edges, surfaces, and depth boundaries (Peterson, 2001). Although a number of theoretical models for object perception have been proposed (e.g., Marr, 1982; Biederman, 1987; Dickinson et al., 1992; Ullman, 2007), the ontogeny of object perception is still not well understood (e.g., Kovács et al., 1999; Hou et al., 2003; Gerhardstein et al., 2004; Hadad et al., 2010a).

Gestalt theorists have proposed that proximity (elements that are close together tend to be grouped together), collinearity or good continuation (elements that are aligned with one another will be grouped into the same contour), common fate (elements that move along the same path likely belong to the same contour), and closure (a closed contour is easier to detect than an open one) are processing heuristics for contour detection and contour integration (Köhler, 1947; Wertheimer, 1958). Within the adult literature, a substantial body of research describing contour perception suggests that contour or edge-level processing reflects the heuristics of proximity, collinearity, common fate and closure (for a review, see Wagemans et al., 2012).

Importantly, the low-level characteristics of natural scenes in the visual world have been shown to be statistically regular; this regularity has been taken as support for the suggestion that Gestalt heuristics may be used for contour detection. Geisler (2008), Geisler and Perry (2009), and Geisler et al. (2001) in particular noted that contours in natural scenes are relatively smooth and therefore heuristics such as proximity and collinearity have a statistical basis in natural scenes. This regularity scaffolds numerous aspects of visual perception including the use of proximity information (Brunswick and Kamiya, 1953), proximity interacting with curvature/collinearity (Geisler et al., 2001; Tversky et al., 2004; Lawlor and Zucker, 2013), figure-ground segmentation (Fowlkes et al., 2007) and closure (for reviews see Kovács, 1996; Pettet et al., 1998; Mathes and Fahle, 2007; Geisler, 2008; Loﬄer, 2008; Geisler and Perry, 2009). Gestalt heuristics therefore take advantage of this natural order. How the mature observer acquires the mechanisms underlying these heuristics, however, is unclear. In nature, proximity and collinearity are highly correlated (Geisler et al., 2001) even in natural scenes in which partial occlusions are frequent (although contrast polarity also plays a role in contour detection in such instances; see Geisler and Perry, 2009).

### CONTOUR PROCESSING – ELEMENTAL DETECTION TO INTEGRATION, IN BRAIN AND BEHAVIOR

The integration of spatially disparate but organizationally related visual information is a fundamental component of object perception, and has been highlighted in the adult psychophysics literature (Field et al., 1993; Kovács and Julesz, 1993; Mathes and Fahle, 2007; for review, see Loﬄer, 2008), the neurophysiological literature (Nelson and Frost, 1985; Ts’o et al., 1986; Gilbert and Wiesel, 1989; Gilbert et al., 1996; Bosking et al., 1997; Li, 1998; Stettler et al., 2002; Cass and Spehar, 2005), and in modeling work (Yen and Finkel, 1998; Grossberg and Williamson, 2001; Voges et al., 2010; Gintautas et al., 2011; Piëch et al., 2013). Following detection of contour segments, integrating these segments into a larger whole, or contour, is generally seen as the next step toward detecting individual objects. While much work has been done on object perception (Johnson, 2001), the present review focuses on low- and intermediate-level studies regarding contour processing to determine the relation between physiology and perceptual capabilities in this domain across development. The next section discusses the lowest level of spatial integration – collinear facilitation in flanker tasks – in terms of physiology and perception. Our discussion then extends up the visual hierarchy, to similarly elucidate larger-scale visuo-spatial integration underpinning higher-order contour processing. Again, this relationship is examined in terms of research from both the psychophysical and physiological perspectives. At its terminus, this section relates the discussed work to higher-level object perception across development.

#### Physiology for elemental detection and integration

The rudiments of object perception begins when light from the visual scene falls on the photoreceptors in the retina. Each photoreceptor detects light from a small fraction of the visual scene. From the photoreceptors, information is sent via ganglion cells to the lateral geniculate nucleus (LGN) and then to area V1 (followed by V2, V3, V4, and V5 via feedforward and feedback connections) in the primary visual cortex. Neurons in area V1 are dedicated to the detection of segments of specific orientations and spatial frequencies (among other visual attributes, Hubel and Wiesel, 1959, 1968; Hubel et al., 1977), referred to as the neuron’s *classical receptive field* (CRF). However, more recent work has shown that neurons in area V1 are also influenced by input from areas outside the CRF. Specifically, detection of a foveated Gabor target (a Gaussian-modulated sinusoidal luminance distribution) is influenced by proximity and collinearity of the flanking elements in a flanker facilitation task (Polat and Sagi, 1993; Shani and Sagi, 2005; Lev and Polat, 2011). When flankers were presented in the 2–6λ range (where λ equals the wavelength of the Gabor itself) and were collinear with the target element, a flanker facilitation effect occurred, reducing the detection threshold for the target element (Polat and Sagi, 1993). This *contextual modulation* of neurons in area V1 can be explained by excitatory and inhibitory long-range horizontal interconnections between neurons. Early reports of the existence of these connections (Rockland and Lund, 1982; Gilbert and Wiesel, 1983, 1989; Nelson and Frost, 1985; Ts’o et al., 1986) have been clearly confirmed (Gilbert et al., 1996; Bosking et al., 1997; Kapadia et al., 2000; Stettler et al., 2002; Gilad et al., 2012). Research suggests that the horizontal connections in the visual cortex underlie at least some Gestalt processes (Field et al., 1993; Kovács and Julesz, 1993; Tversky et al., 2004; Mathes and Fahle, 2007; for review, see Loﬄer, 2008). Information detected by neurons in area V1 must, however, be integrated into more global-level contours that can be used to detect objects and subsequently, form a meaningful percept of the visual scene.

Two complimentary, but computationally quite opposite processes appear to occur via these connections in V1 (and perhaps in V2; Polat, 2013). The first is a process of object boundary detection supported by iso-orientation inhibition, whereby cortical columns sensitive to a particular orientation inhibit nearby regions sharing orientation information. This inhibition occurs less at object edges than inside or outside these boundaries, making the enclosing regions of the visual field that denote objects explicit and salient. This process appears to occur early, and does not appear to require top-down input to operate, functioning instead as part of an initial bottom-up process. The second is a process of attention-mediated region-filling, whereby regions sharing orientation information propagate an excitatory signal that fills in textures and stops at object boundaries (similar to classical grassfire algorithms, e.g., Blum, 1967; Kovács et al., 1998). This process appears to occur following the boundary detection process, and indeed may depend on it, as the boundaries discovered in the first process designate for the second process which regions of the visual field need filling in. Anatomically, superficial layers in V1 columns receive feed-forward inputs and perform pre-attentive boundary detection, whereas region filling appears to be triggered in deeper layers (layers IV and V) as a function of top-down attentional feedback from higher layers (Polat, 2013).

The physiology supporting a mechanism for contour detection appears to be present early in infancy, at least in a rudimentary form (see Burkhalter et al., 1993; Kovács et al., 1999; Gerhardstein et al., 2004; Hadad et al., 2010a). Using human brains ranging from 24 weeks gestational age to those of children up to 5 years of age, Burkhalter et al. (1993) documented that the basic structures of V1 in the primary visual cortex are in place early in life. However, the vertical connections between layers and horizontal connections within layers of the visual cortex show protracted development. Specifically, Burkhalter et al. (1993) describe a dense network of horizontal connections that first emerges prenatally around 37 weeks gestation. The patchiness characteristic of the horizontal connections in adults (Gilbert et al., 1996; Stettler et al., 2002) begins emerging at 7 weeks post-natal and is anatomically “adult-like” by 24 months (Burkhalter et al., 1993; also see Galuske and Singer, 1996 for a similar description of the development of horizontal connections in cats). Computational models of development in the visual system strongly suggest that the spatial distribution of horizontal connections in the cortex can arise from self-organization following visual input (Voges et al., 2010) and from processing “real” images (Prodöhl et al., 2003). For example, Grossberg and Williamson (2001) implemented a (modeled) period of exuberant growth and a period of refinement for horizontal connections following initial visual input by emphasizing the role of balance between excitation and inhibition. Similarly, Choe (2001) demonstrated that these horizontal connections link columns whose orientations are collinear, and that the connection statistics match the edge co-occurrence statistics in natural scenes (Geisler et al., 2001). It appears, therefore, that considerable visual development occurs during the postnatal period, including the development of contour detection capabilities.

#### Perceiving contours embedded in noise

Prior to beginning our review of the influence of Gestalt principles on element detection and contour integration, we first present a summary of approaches and stimuli used in the more recently emergent literature investigating these questions. When perceiving natural scenes, contours must be detected despite the high degree of visual noise obscuring the signal at the retina. For example, within natural scenes such as a field of flowers there are typically multiple overlapping contours referring to multiple different objects, patterns or depth information. Careful psychophysical methods analogous to this signal extraction problem have been developed using Gabor patch contours embedded in noise. Gabor elements are ideal stimuli with which to measure contour detection in the visual system since the Gabor elements model the orientation selective cells in V1. Perception of a contour composed of Gabor elements relies on the long-range horizontal connections between these orientation selective cells. Using Gabor patches to study contour detection visual noise is done by manipulating relative noise density, or the ratio of the density (*D*) of surrounding noise elements over the density of elements on a contour. For example, *D* = 1.0 means that the density of elements on the contour matches that of the noise elements, while *D* < 1.0 means that the density of the contour elements is less than those on the contour and *D* > 1.0 means that the density of the contour is greater than the density of the noise. Adult participants are relatively good at detecting contours embedded in noise, the minimum noise density ratio at which a contour can still be detected is *D* = 0.67 (Kovács et al., 1999).

**FIGURE 1:**
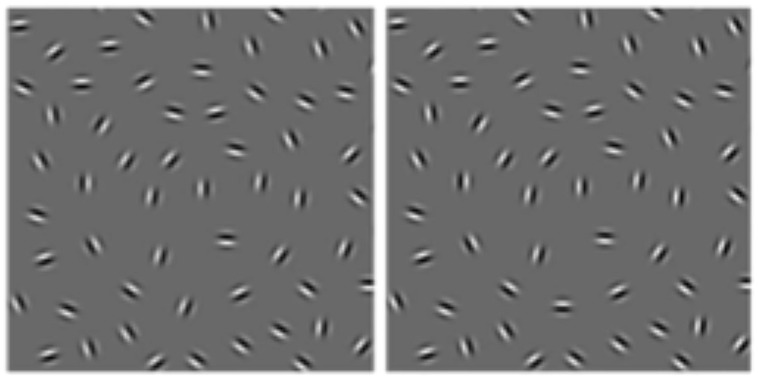
**Example of the open (left) and closed (right) contours composed of Gabor elements used in Gerhardstein et al. (2004) at *D* = 0.09**.

Developmental work has started to document contour detection thresholds, and thus the functionality of long-range horizontal connectivity, in children. Using a mobile conjugate reinforcement procedure in which infants learn to kick to move a mobile consisting of three cards displaying either Gabor contours embedded in noise or only noise (e.g., circle vs. noise), Gerhardstein et al. (2004) assessed contour detection in 3-month old infants (see **Figure 1**). Infants were trained with one stimulus and tested with the other 24 h after training; baseline kick rate in response to the (new) test stimulus was taken as evidence that infants could discriminate between the two. Gerhardstein et al. (2004) found that for circular contours, at 3-months of age *D* = 0.9 was the minimum noise density ratio for contour detection. In other words, infant kick rate was greater than baseline in the immediate test, demonstrating that the infants could discriminate the stimulus from noise and no different from baseline in the discrimination test 24 h later demonstrating that the infants could discriminate between the stimuli. The applicability of the mobile conjugate reinforcement procedure for studying contour detection across older ranges of development, however, is limited.

Alternative procedures have been developed to study contour detection abilities across development. Baker et al. (2008) used a visual expectation cueing paradigm and an eye-tracker to assess detection in 6-month old infants, in a procedure in which the presentation of a square composed of Gabor elements predicted the subsequent appearance of a target on one side of the screen and a circle composed of Gabor elements predicted the subsequent appearance of a target on the other side of the screen. Predictive (anticipatory) looks to the correct side for the target stimulus following the presentation of the contour (square vs. circle) were evidence that infants could detect and discriminate between the contours. Overall, Baker et al. (2008) found that 6-month-old infants could accurately detect and discriminate the shape of a contour embedded in noise only when the noise density ratio was *D* = 0.90 or higher, similar to 3-month-olds, suggesting that little functional development of this ability takes place in the first 6 months.

Research with older children and adults suggests that noise density continues to play a role in contour detection across a much longer range of development (Kovács et al., 1999; see also Benedek et al., 2010). When participants are asked to point at a contour presented in a Gabor patch on a card held in front of them, the minimum noise density ratio at which a contour can still be detected is *D* = 0.84 at 5–6 years, *D* = 0.70 at 13–14 years and *D* = 0.67 into adulthood (Kovács et al., 1999). In contrast, when the Gabor patch contours were presented on a computer screen until the participant responded or for a maximum of 15 s, Hipp et al. (2014) found that children under 9 years of age could not perform above a 75% accuracy threshold at noise density ratios of *D* = 0.90. Importantly, Kovács et al. (1999) determined the minimum *D* for each age group by the last correctly identified card, while Hipp et al. (2014) used a more conservative threshold measure of responding correctly 75% of the time to a given *D* to control for chance. Nevertheless, noise density plays an important role in contour detection during development and the tolerance for noise density when detecting contours increases across development.

#### Gestalt principles for elemental detection and integration

Separating the proximity and collinearity principles functionally is difficult by some definitions. Indeed, it may be prudent to consider them as aspects of a single description of the relation between two or more parts of the visual scene. Given this, it is perhaps no surprise that much of the behavioral research on perceptual grouping manipulates both proximity and collinearity. Following the work on flanker facilitation (e.g., Polat and Sagi, 1993), the role of collinearity in contour integration has been determined by *jittering* Gabor elements along a contour (Field et al., 1993) as well as through the use of noise manipulations. Jitter refers to a manipulation in which a contour is first rendered using co-aligned, identical Gabor elements that fall on a (typically curved) path embedded in noise (Gabor elements of the same spatial frequency and phase, but random orientation and position). Elements on the contour are then jittered by a manipulated amount in a random direction, to reduce the extent to which contour elements follow the true path of the contour, and the level of such jitter at which detection ceases is the threshold. Field et al. (1993) found that adult contour detection dropped off rapidly after about 15^∘^ of orientation disparity between elements, suggesting that the greater the collinearity from element to element on the contours, the more easily they were detected from a field of random noise elements. Similarly, participants can perform contour detection even over the relatively large inter-element distances of 0.9^∘^ (Field et al., 1993), suggesting that spatial integration can occur over large areas of the visual cortex. Indeed, the long-range horizontal interconnections between neurons span cortical distances of up to 8 mm (Gilbert et al., 1996). Overall, contours are easier to detect from a background of randomly oriented noise elements of the same size and shape if elements are proximal and coaligned elements (Field et al., 1993; for a more recent example see also Beaudot and Mullen, 2001).

Early in development, proximity between the elements on a contour plays a larger role in determining the detectability of the contour. Using Gabor stimuli, Hipp et al. (2014) noted that when inter-element spacing was 9λ (which is quite far apart, such that spacing is analogous to object contours that are partly occluded in the visual scene) 7–9 year olds only detected contours when *D* = 1.00, and 5–6 year old children failed to detect the contour reliably even at that level. However, when the inter-element spacing was reduced from 9 to 4.5λ, 7–9 year olds performance was nearly adult-like, and 5–6 year old children were able to detect the contour at the *D* = 0.90 level. Performance was also improved even in 3–4 year olds, who improved from not being able to detect the contour at all to being able to detect the contour at *D* = 1.0 at 4.5λ. In other words, doubling proximity while keeping relative noise ratio constant dramatically improved performance across a broad span of developmental time. Importantly, in adults the noise density tolerated for contour detection is relatively independent of the proximity between elements (Kovács et al., 1999).

Other research in developmental psychophysics investigating the use of local heuristics in contour detection supports the adult data, and suggests that the effects of collinearity and proximity are not independent. Hadad et al. (2010a) measured the ability to detect an egg-shaped contour constructed of Gabor elements by adults and children aged 7–14 years. Overall, adults and older children demonstrated a higher tolerance for noise density as collinearity increased, while proximity played more of a role when collinearity decreased (increased jitter between contour elements). In contrast, in 7-year-old children both proximity and collinearity play a significant role such that even when collinearity is high, children were hindered by low proximity. By 14 years of age, children rely less on proximity when collinearity is high, but are not yet adult-like. Notably, greater reliance on proximity for contour integration early in childhood may reflect functionally shorter-range horizontal connections early in development (for a similar argument, see Kovács et al., 1999; Kovács, 2000; Hipp et al., 2014). If so, it may be the case that the protracted development of contour integration is potentially sourced in the extended development of this aspect of the physiology of the visual system (see also Benedek et al., 2010).

It appears, then, that developing humans acquire correlations in orientation information (i.e., collinearity) within a limited spatial extent around a particular location (i.e., proximity). This spatial extent appears to expand with age and experience. The development of these proximity and collinearity heuristics in the visual system is suggestive of developmental statistical learning, progressing at a rate that depends on the robustness of the natural correlations that support it. Indeed, Geisler et al. (2001) and Geisler and Perry (2009) documented the edge co-occurrence statistics in natural scenes which suggested that, in natural scenes, the rate at which edge elements share orientation drops off rapidly with distance from a target. Behaviorally, Hall et al. (2010) reported increased detectability for targets whose temporal presentation sequence mirrored statistical regularities as outlined by Geisler et al. (2001). That is, collinearity in nature weakens with increased spatial-temporal distance.

The use of proximity and collinearity heuristics for contour detection and integration appear to have different developmental trajectories. The use of proximity information appears to begin early in development (Hipp et al., 2014). However, the distances required for successful detection and the noise levels tolerated are greatly reduced in infants and children compared to adults, and develop gradually throughout ontogeny (Hipp et al., 2014). In contrast, the use of proximity information appears to begin later on in childhood (e.g., Hadad et al., 2010a). With respect to the physiological development of the visual system, these results support the neurophysiological data suggesting significant developments in axonal lengths and neuron density facilitating the development of long-range horizontal connections in V1 occurring across the first several years of life (Burkhalter et al., 1993). Moreover, it may be that these studies also index the development of horizontal connectivity in V2, where receptive field sizes are greater, but this remains an open question.

#### Physiology for higher-order contour integration

Although the processes fundamental to spatial integration of disparate contour elements likely occur in V1 (Polat, 2013), recent research suggests that the likely cortical site of larger-scale contour representation is V2 (Huang et al., 2006) indicating that these integrative processes might scale with receptive field size. The proximity and collinearity effects found in flanker facilitation tasks extend to larger-scale contour integration (Polat, 1999; Polat and Bonneh, 2000; Cass and Spehar, 2005; Zhaoping, 2011), such that elements are grouped into contours if they share orientation information and are sufficiently close together (see Geisler et al., 2001). Like V1, excitatory and inhibitory long-range horizontal connections in area V2 are likely to be the physiological source for the implementation of a contour integration mechanism and are invoked by multiple models of contour integration in vision (e.g., Li, 1998, 2002; Yen and Finkel, 1998; Usher et al., 1999; Gintautas et al., 2011; Zhaoping, 2011; Piëch et al., 2013).

Evidence of differential processing of lower-level properties and higher-level properties in the visual system has been demonstrated using a monoptic/dichoptic masking procedure to test adult participants for perceptual after-effects of closed and open contours (Sweeny et al., 2011). Monoptic masking is known to disrupt lower-level visual processing and spare higher-order processing, while dichoptic masking affects processing in the opposite way. Sweeny et al. (2011) found closed contour after-effects were evoked following monoptic, but not dichoptic masking, while the opposite pattern was found for open contours. This result supports the idea that contour integration via a closure mechanism is implemented in visual areas beyond V1 in the pathway. Specifically, implementation of the global closure heuristic during visual processing likely occurs in either area V2, thought to be the site of global contour integration (Huang et al., 2006), or area V4, which performs population coding of shape (Pasupathy and Connor, 2002). Nevertheless, long-range connections within and between cortical sites provide a mechanism through which the input from several receptive fields can interact and bind together spatially disparate segments of a contour using a global closure heuristic. Neural synchrony resulting from the oscillation of these excitatory neurons is argued to be the binding mechanism (Kovács, 1996; Yen et al., 1998; Sweeny et al., 2011; see also Gilad et al., 2013). The idea is that a reciprocal relation exists between the strength of neural synchrony and the salience of the contours. Global closure may therefore influence local level feature enhancement in a top-down fashion (Mathes and Fahle, 2007).

In adults, a delicate balance between neural synchrony-mediated excitation and surround suppression-mediated inhibition controls the characteristics of local and global contextual modulation found in various perceptual grouping tasks (Yen and Finkel, 1998). This design inherently requires neural responses to balance the involvement of excitatory and inhibitory circuits simultaneously (Grossberg and Williamson, 2001). Developmentally, acquiring this essential balance is critical for flexible perceptual learning and achievement of reliable perceptual grouping in adulthood (Grossberg and Williamson, 2001; Pinto et al., 2010). One mechanism responsible for achieving balance in neural synchrony is GABAergic expression responsible for local inhibition in the visual cortex, which is known to develop throughout the lifespan (Pinto et al., 2010). This inhibition is thought to underpin the oppositely signed surround portion of the oriented center-surround receptive fields in early visual cortex. This GABAergic expression undergoes three “main transition stages” in which rapid switches in GABAergic signaling in visual cortex occur – one in early childhood, another in early teenage years and yet another as signs of aging commence (Pinto et al., 2010). Given the developmental psychophysics research described above, it seems likely that similar developmental neurochemical foundations underlie the development of excitatory circuits.

#### Gestalt principles for higher-order contour integration

Closure represents a global heuristic for contour integration, depending on the higher-order pattern of relations between more than two elements. Psychophysical studies show that adults exhibit a *closure superiority effect*; that is, detectability of closed figures is enhanced relative to open figures (Kovács and Julesz, 1993; Mathes and Fahle, 2007; Machilsen and Wagemans, 2011; Gerhardstein et al., 2012). For instance, using a contour detection task with adults, Kovács and Julesz (1993) incrementally added co-aligned elements to a circular contour and found that performance was not enhanced until the contour was closed. Closure therefore elicited a pop-out effect, by their interpretation. While there has been some contention regarding whether a global heuristic such as closure needs to be invoked to explain the closure superiority effect (Tversky et al., 2004), recent research (Gerhardstein et al., 2012) strongly suggests that such a mechanism does operate in the visual system. By separately manipulating collinearity and closure using circles and S contours, Gerhardstein et al. (2012) showed that closure enhances detectability of a contour separate from local grouping heuristics.

Closure facilitates contour integration (Pettet et al., 1998; Mathes and Fahle, 2007; Gerhardstein et al., 2012), object detection (Machilsen and Wagemans, 2011), texture-segmentation (Atkinson and Braddick, 1992; Norcia et al., 2005; Machilsen and Wagemans, 2011), and figure-ground segmentation (Field et al., 1993; Kovács and Julesz, 1993; Kovács, 1996). To date, few studies have explored the development of such a closure mechanism across childhood (Gerhardstein et al., 2004; Hadad and Kimchi, 2006; Baker et al., 2008; Hadad et al., 2010a; Hipp et al., 2014). Using a mobile conjugate reinforcement procedure, Gerhardstein et al. (2004) found that unlike adults, 3- to 4-month-old infants show no evidence of a closure superiority effect when detecting contours embedded in noise regardless of noise density; manipulation of contour type (open or closed) did not affect sensitivity to the contour at this age. Moreover, at 3–9 years of age children appear to use the local proximity heuristic rather than closure when detecting closed and open contours composed of Gabor elements and embedded in noise (Hipp et al., 2014). Specifically, children failed to show a closure superiority effect at 4.5λ or 9λ, although overall contour detection performance was better when proximity was 4.5λ rather than 9λ. Adults, in contrast, demonstrated a closure superiority effect at both 4.5 and 9λ and at the highest noise density level, *D* = 0.80. Thus, the presence of closure information for contour integration does not appear to compensate for children’s dependence on proximity information early in development.

The interaction between the local heuristics of proximity and collinearity and the global closure heuristic appears to change across development. Using a different procedure, Hadad and Kimchi (2006) tested children aged 5 and 10 years and adults on their ability to detect a concave shape embedded among convex shapes in a visual display. The shapes were composed of disconnected line segments that were either open or closed. Notably, this procedure was a visual search task to determine the role of closure on visual search efficiency. Overall, performance by 10 year old children and adults was unaffected by changes in proximity when closure and collinearity information was available. However, at 5 years of age, children’s concave contour detection performance was affected by decreasing proximity between line segments regardless of whether closure alone or closure and collinearity information was available. Overall, research with children suggests that a closure mechanism may not function at adult levels until into adolescence (e.g., Pennefather et al., 1999). Thus, it appears that the global closure mechanism also undergoes a prolonged developmental trajectory, gradually evoked and tuned across childhood and into adolescence. In sum, the reviewed psychophysics data (Kovács et al., 1999; Gerhardstein et al., 2004; Hadad and Kimchi, 2006; Hadad et al., 2010a; Hipp et al., 2014) suggest an extended developmental trajectory of the visual system that may be explained by physiological development (e.g., Burkhalter et al., 1993).

This interaction between proximity and collinearity also affects perception of the illusory contours formed by Kanizsa squares. To perceive the illusory contour created by Kanizsa elements, the perceiver needs to bind the Kanizsa elements into an object contour by filling in the gaps of the Kanizsa elements. It is perhaps not surprising that although when bound together into an illusory contour, the elements form a closed contour, the proximity heuristic is particularly important. Proximity within Kanizsa squares is defined by a support ratio, the length of the contour specified by the Kanizsa elements to the total length of the illusory contour. Higher support ratios typically result in stronger illusory contour perception given that the observer must traverse a smaller gap to perceive the contour. For example, Watanabe and Oyama (1988) found that Kanizsa illusory squares were perceived as stronger (e.g., greater contrast and clarity) when proximity between the four elements was high (see also Shipley and Kellman, 1992; Hadad et al., 2010b). Indeed, 4-month old infants perceive an illusory contour formed by a Kanizsa square as an occluding object only when proximity was high and the square formed a narrow occluder (Bremner et al., 2012). Thus, the greater dependence upon the proximity heuristic for illusory contours is may reflect limitations in the distance projected by the horizontal connections in the visual system.

Within the context of whole object perception, for young infants, contour integration may be achieved by a greater reliance on the grouping heuristic of common fate. Indeed, sensitivity to motion develops around 3- to 4-months and may provide a scaffold for the use of proximity and collinearity heuristics in later infancy (Johnson and Aslin, 1996, 1998; Smith et al., 2003; Johnson et al., 2012). Using occluded objects on a textured background, Johnson and Aslin (1996, 1998), Smith et al. (2003), and Johnson et al. (2012) found that 3- to 4-month old infants could perceive object unity when the two visible portions of an object were moving together. In contrast, when there was no motion information available infants did not perceive object unity for a partly occluded object (Kellman and Spelke, 1983). Importantly, common motion is not the sole factor for perceiving object unity when objects are partly occluded. For example, Johnson (2004) found that infants were better able to perceive object unity when the occluding object was narrow, compared to a wide occluding object. The early use role of motion for contour integration consistent with the earlier development of the M-pathway in the infant visual system compared to the horizontal connections (Burkhalter et al., 1993).

## FUTURE DIRECTIONS

Taken together, the findings discussed in the present review inform research on the development of object perception in a number of ways. With respect to distinguishing a stationary object from the background, the principles of proximity (which will likely be high if the object is not occluded), collinearity (depending upon the object’s shape), and the emergent property of closure all appear to play a role. Moreover, according to the research reviewed (e.g., Johnson et al., 2012), for infants, a moving object is clearly easier to segment from the background than a static object, demonstrating the importance of the motion-based “common fate” heuristic. Importantly, the research in the present review informs the development of bottom-up processes for object perception and does not consider the role of top-down processes (e.g., Needham et al., 2005; for review, see Quinn and Bhatt, 2009), although as with the development of horizontal connections, physiological findings also suggest a protracted development of feedback connections in the visual system (Burkhalter, 1993). However, many studies on object perception lack the low-level control employed in the contour detection and integration psychophysics studies discussed in the present review, for example controlling color, background noise, brightness, and depth cues. Thus, to more accurately map the findings discussed here onto those investigating the development of object perception, a set of studies marrying the methods of the lower-level psychophysics studies with higher-level object perception investigations would be informative.

Within the psychophysics literature on contour detection and integration, developmental studies are relatively sparse and as such, there has been very little systematic documentation on the development of these abilities. The role of noise density on contour detection when stimuli are composed of Gabor elements has been systematically studied, documenting a progressive increase in the tolerance for noise elements across development and into adulthood (Kovács et al., 1999; Gerhardstein et al., 2004; Baker et al., 2008; Hipp et al., 2014). The use of Gestalt heuristics for contour detection across development, however, has not been documented systematically. For example, studies investigating the use of the closure heuristic leap from investigating 3- to 4-month old infants (Gerhardstein et al., 2004) to 3–9 year old children (Hipp et al., 2014). Additionally, studies investigating proximity begin with investigation of children from 3 to 4 years (Kovács et al., 1999; Hipp et al., 2014) and studies investigating collinearity start with investigation of children at 7 years of age (Hadad et al., 2010a). Moreover, the terms “contour detection” and “contour integration” have been used to refer to a number of different tasks from detecting contours composed of Gabor elements (e.g., Baker et al., 2008; Hadad et al., 2010a; Hipp et al., 2014), illusory contours using Kanizsa squares (e.g., Hadad et al., 2010b) and a visual display of concave and convex shapes (e.g., Hadad and Kimchi, 2006). While each task clearly calls upon the long-range horizontal connections in the visual system, a systematic investigation considering the differences between the tasks is needed. Future work should focus on a systematic study of the development of contour detection across development from infancy and childhood, through adolescence and into adulthood.

By systematically tracking the development of the visual system from functional onset early in infancy to adult-level functioning in adolescence and into adulthood, we can begin to infer how the visual system continues to develop physiologically. Eye tracking methodology may provide one means by which the development of contour detection can be systematically documented given that this method can be used across development (e.g., Taylor and Herbert, 2014). Furthermore, although it is clear that contour detection occurs early on in the visual system (e.g., Huang et al., 2006), it is not possible to conclude whether the majority of the contour detection mechanisms are implemented in V1 or in V2, a region containing cells with a larger receptive field (e.g., Smith et al., 2001).

## CONCLUSION

While the visual system appears to be functional early on in development, it is clear from the present review that adult-level functionality does not begin to emerge until late in childhood and early adolescence (Kovács et al., 1999; Gerhardstein et al., 2004; Hadad and Kimchi, 2006; Hadad et al., 2010a, b; Hipp et al., 2014). Specifically, Burkhalter et al. (1993) note that the patchiness characteristic of the horizontal connections is anatomically “adult-like” by 24-months (also see Burkhalter, 1993). In contrast, psychophysics data demonstrates that while 3- to 6-month old infants are capable of detecting contours embedded in noise (e.g., Gerhardstein et al., 2004; Baker et al., 2008), the use of proximity, collinearity and closure information apparently does not become adult-like until preadolescence or later (e.g., Kovács et al., 1999; Hadad and Kimchi, 2006; Hadad et al., 2010a; Hipp et al., 2014). Thus, the developmental time course for physiology and psychophysics appear to differ considerably but nonetheless suggest a protracted development for contour processing.

The difference between functional physiological development of the visual system in childhood and a functionally mature physiological visual system in adulthood may explain the disparity between behavioral and physiological data. In addition, the extended physiological development of the visual system may be related to the extent and features of the visual input (see Gilbert et al., 2001). For example, by exploiting congenital cataract, Maurer et al. (1999) found that visual acuity begins developing within the first hour of receiving visual input, but not before. Importantly, in adulthood, short exposure to visual input that includes edges with orthogonal alignments facilitates orthogonal contour detection as mediated by changes in the neural representation (Schwarzkopf et al., 2009). Visual input therefore remains an important tool for mediating contour detection in the visual system (Gilbert et al., 2001; Sagi, 2011) and may account in part for the protracted development of the visual system.

To conclude, contour detection appears to become increasingly sensitive to long-range correlations in the visual world as development proceeds, with the eventual magnitude of this span not fully realized until at least adolescence. Physiologically, ontogeny is likely characterized by increases in efficiency of the plexus of horizontal connectivity connecting cortical columns in V1 and V2 in the visual cortex. This intrinsic connectivity thus becomes increasingly effective at integrating representations over greater and greater cortical distances as expertise with short-range pairings based on orientation is achieved. This process likely proceeds into adulthood, as experience is gleaned with less common – but still robust – longer-range correlations present in nature.

## Conflict of Interest Statement

The authors declare that the research was conducted in the absence of any commercial or financial relationships that could be construed as a potential conflict of interest.
